# Dietary Polycan, a β‐glucan originating from *Aureobasidium pullulans* SM‐2001, attenuates high‐fat‐diet‐induced intestinal barrier damage in obese mice by modulating gut microbiota dysbiosis

**DOI:** 10.1002/fsn3.4235

**Published:** 2024-05-26

**Authors:** Gwang‐Pyo Ko, Tatsuya Unno, Young‐Suk Kim, Jungman Kim

**Affiliations:** ^1^ Faculty of Biotechnology, School of Life Sciences SARI Jeju National University Jeju Korea; ^2^ Department of Microbiology Chungbuk National University Cheongju Korea; ^3^ Glucan Co. Ltd. Jinju Korea; ^4^ Subtropical/Tropical Organism Gene Bank Jeju National University Jeju Korea; ^5^ Jeju Institute of Korean Medicine Jeju Korea

**Keywords:** dysbiosis, gut microbiota, high‐fat diet, Polycan, tight junction

## Abstract

Various metabolic diseases caused by a high‐fat diet (HFD) are closely related to gut microbiota dysbiosis and epithelial barrier dysfunction. Polycan, a type of β‐glucan, is effective in treating anti‐obesity and metabolic diseases caused by HFD. However, the effect of Polycan on dysbiosis and epithelial barrier damage is still unknown. In this study, the effects of Polycan on dysbiosis and intestinal barrier damage were investigated using HFD‐induced obese model mice. C57BL/6 mice were fed a HFD for 12 weeks and treated with two different doses of Polycan (250 and 500 mg/kg) orally administered during weeks 9 to 12. Polycan supplementation increased the expression of tight junction genes (zonula occludens‐1, occludin, and claudin‐3) and short‐chain fatty acid (SCFA) content while reducing toxic substances (phenol, *p*‐cresol, and skatole). Most significantly, Polycan enriched SCFA‐producing bacteria (i.e., *Phocaeicola*, *Bacteroides*, *Faecalibaculum*, *Oscillibacter*, Lachnospiraceae, and Muribaculaceae), and decreased the Firmicutes/Bacteroidetes ratio and toxic substances‐producing bacteria (i.e., *Olsenella*, *Clostridium* XVIII, and *Schaedlerella*). Furthermore, microbial functional capacity prediction of the gut microbiota revealed that Polycan enriched many SCFA‐related KEGG enzymes while toxic substance‐related KEGG enzymes were depleted. These findings indicated that Polycan has the potential to alleviate HFD‐induced intestinal barrier damage by modulating the function and composition of the gut microbiota.

## Graphical abstract

A high‐fat diet is a major cause of intestinal barrier dysfunction and gut microbiota dysbiosis. In this study, we examined the mitigative effect of Polycan, a type of β‐glucan extracted from Aureobasidium pullulans SM‐2001, on high‐fat diet‐induced intestinal barrier dysfunction and dysbiosis in mice.

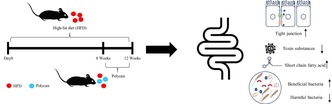

## 1 INTRODUCTION

The intestinal mucosa is the major interface that blocks ingested harmful elements, such as bacteria, viruses, and toxic substances, from entering and causing harm to the body. An impaired intestinal barrier allows the passage of luminal pathogens. Furthermore, intestinal barrier impairment is closely associated with the development of metabolic diseases (Konig et al., 2016). High‐fat diets (HFDs), particularly, damage the intestinal barrier and consequently increase intestinal permeability, which increases the risk of metabolic diseases, such as obesity, inflammatory bowel disease, nonalcoholic fatty liver disease, and diabetes (Di Tommaso et al., 2021; Rohr et al., 2020; Sellmann et al., 2015). Intestinal barrier function is regulated by tight junction (TJ) proteins consisting of zonula occludens (ZO), occludins (OCLDN), and claudins (CLDN). If the expression of these proteins is insufficient or downregulated, the intestine becomes severely leaky (Bischoff et al., 2014). Therefore, the expression of these proteins is vital to maintaining intestinal barrier integrity and function.

Accumulated data have shown that intestinal barrier damage is associated with gut microbiota changes. The gut microbiota digests food and produces metabolites. When indigestible polysaccharides are ingested, short‐chain fatty acids (SCFA) are synthesized by intestinal microorganisms. These metabolites significantly contribute to maintaining intestinal barrier integrity and the energy (food) intake–expenditure balance (Liu et al., 2021; Ridaura et al., 2013). HFDs are factors of dysbiosis that inhibit SCFA production, which in turn can impair intestinal barrier function and increase intestinal permeability (Murphy et al., 2015; van der Hee & Wells, 2021). Conversely, undigested proteins are fermented by the gut microbiota, potentially generating harmful phenolic and indolic substances (Smith & Macfarlane, 1997a). Phenolic compounds are derived from tyrosine, whereas indolic compounds are derived from tryptophan (Smith & Macfarlane, 1997b). Previous studies have shown that these toxic substances can reduce and damage intestinal epithelial barrier function (Cerini et al., 2004; Kurata et al., 2019; McCall et al., 2009).

In recent years, much attention has been focused on the efficacy of natural products or extracts in improving dysbiosis and intestinal damage. Phenolic compounds from noni fruit, polyphenols from kiwifruit, and many other plant extracts have been found to improve dysbiosis and strengthen the intestinal barrier in animal models (Lee et al., 2022; Li et al., 2022; Wang et al., 2023; Yuan et al., 2021). β‐glucans are naturally occurring nondigestible and soluble dietary fibers found in grains, fungi, yeasts, and bacteria. It is nonresistant, strengthens immunity, and exhibits anti‐inflammatory, anti‐cancer, anti‐obesity, and other physiological activities (Bai et al., 2019; Ciecierska et al., 2019). Previous studies have shown that β‐glucans not only improved dysbiosis induced by a low‐fiber diet but also improved dysbiosis and barrier dysfunction induced by HFD (Mo et al., 2022; Muthuramalingam et al., 2019).

Polycan is a type of β‐glucan extracted from the black yeast *Aureobasidium pullulans* SM‐2001. In addition, Polycan, which includes anti‐hyperlipidemia (Lim et al., 2015) and improvement of bone formation (Jung et al., 2016), has been included in an individually approved type of ingredient for healthy functional food that is certificated by the Korea Food and Drug Administration (KFDA) and Generally Recognized As Safe (FDA GRAS). However, the effects of Polycan on gut dysbiosis, microbial metabolites, and intestinal damage in obese mice are unclear. Therefore, this study aims to determine whether Polycan ameliorates intestinal damage in obese mice with gut dysbiosis by modulating the gut microbiota and microbial metabolites.

## 2 MATERIALS AND METHODS

### 2.1 Polycan preparation

Polycan used in this study was provided by Glucan Co. Ltd. (Jinju, Korea). It is a β‐glucan fermented using *Aureobasidium pullulans* SM‐2001 (average molecular weight: 2.6 × 10^5^ Da; β‐1,3/1,6‐glucan) (Seo et al., 2002).

### 2.2 Animal experimental design

5‐week‐old male C57BL/6J mice were purchased from Damool Science Inc. (Daejeon, Korea). The breeding room environment was maintained in a light–dark cycle of 12 h at a temperature of 22°C ± 2°C with 50% ± 5% relative humidity. The mice were provided ad libitum access to both food and water. Following a one‐week acclimation period, they were randomly divided into five groups, each consisting of 9 mice: a control group (ND; normal diet); a model group (HFD; high‐fat diet); a positive control group (GOS; high‐fat diet with galacto‐oligosaccharide, 500 mg/kg); a low‐dose Polycan group (Polycan250; high‐fat diet with Polycan, 250 mg/kg); and a high‐dose Polycan group (Polycan500; high‐fat diet with Polycan, 500 mg/kg). The mouse feed was purchased from Toto Bio Co., Ltd. (Gyeonggi‐do, Korea), and its ingredients and proportions are shown in Table S1.

To induce dysbiosis in HFD, no treatments were performed during the first 8 weeks. After 8 weeks, the mice were orally administered with GOS, Polycan (GOS, Polycan250, and Polycan500 groups), or the same volume of RO water (ND and HFD groups) for 4 weeks daily. Body weight was recorded once a week, and food intake was measured daily for each cage in the week preceding the sacrifice. At the end of the experiment, fresh feces samples were collected to analyze the microbial community and metabolites. The mice were sacrificed by ether inhalation, after which liver tissue, testicular fat, and colon tissue samples were collected.

### 2.3 Histological analysis

Colon tissue was fixed in 10% buffered‐neutral formalin and then washed with distilled water to remove the fixative fluid. The colon tissues were sectioned into 4 μm‐thick sections and processed with hematoxylin and eosin (H&E) staining to evaluate histological injury (Histoire, Korea). Histological changes were examined by light microscopy (100×). Colon histologic score was assessed following a published scoring system and shown in Table S2 (Dieleman et al., 1998; Luo et al., 2024).

### 2.4 Analysis of tight junction mRNA expression

Total RNA was extracted from frozen colon tissue using the RNAiso Plus reagent (TaKaRa Bio. Inc., Shiga, Japan). Then, 1 μg of RNA was reverse‐transcribed into cDNA using the PrimeScript™ 1st strand cDNA Synthesis Kit (Takara Bio Inc.). Real‐time PCR (Thermal Cycler Dice® Real Time System Lite; Takara Bio Inc.) was conducted using TB Green™ Premix Ex Taq™ (Takara Bio Inc.) for the quantitative analysis of TJ genes. The primer sequences utilized in this study are outlined in Table S3.

### 2.5 Measurement of microbial metabolites

The concentrations of SCFAs (acetate, propionate, and butyrate) and toxic substances (phenol, *p*‐cresol, and skatole) were measured using gas chromatography (GC; Nexis GC‐2030, Shimadzu, Japan) with a flame ionization detector (FID). 50 mg of each fecal sample was homogenized with 450 μL of absolute methanol using a vortexer for 20 min. To extract SCFAs, the mixture was adjusted to pH 2–3, and the reaction was performed at room temperature for 10 min with repeated homogenization every 3 min. However, this process was not performed for the extraction of toxic substances. The mixture was centrifuged at 4°C at 9,358 *g* for 5 min, and the supernatant was filtered through a 0.45 μm pore membrane. Each GC column's information and analysis conditions are shown in Table S4.

### 2.6 Microbial community changes

Genomic DNA was extracted from the feces using the QIAamp PowerFecal Pro DNA Kit (Qiagen, Germantown, MD, USA). To analyze the fecal microbial community, we constructed a MiSeq library. Two‐step PCR was used, and briefly, the V3‐V4 hypervariable region of the 16S rRNA gene was amplified and index primers were attached. The PCR conditions are shown in Table S5. All purification steps were performed using the HiAccubead DNA purification system (AccuGene, Incheon, Korea) following each PCR. Finally, Miseq library constructs from each sample were pooled at the same concentration. Sequencing was performed using the Miseq sequencing platform at Macrogen Inc. (Seoul, Korea).

The raw data from 16S rRNA sequencing on the MiSeq platform were analyzed using MOTHUR software (Schloss et al., 2009). Raw data sequencing was conducted by paired‐end assembly and aligned with SILVA Database version 138 (Quast et al., 2013). To detect and remove rare, singleton, and chimeric sequences, “pre.cluster,” “split.abund,” and “chimera.vserach” subroutines were used (Rognes et al., 2016). The bacterial taxonomic classification was carried out using version 18 of the Ribosome Database Project (Cole et al., 2007). Sequences other than bacteria (i.e., chloroplast‐mitochondria‐unknown‐Eukaryota) were eliminated. Sequencing results were clustered and assigned as operational taxonomic units (OTUs) at 97% similarity using OptiClust (Westcott & Schloss, 2017). For further downstream analyses, the number of reads was normalized to 9715 size reads. Alpha diversity was calculated based on Chao and Shannon. The dissimilarity between each group was assessed using Bray–Curtis dissimilarity coefficients (Beals, 1984) and visualized through nonmetric multidimensional scaling (NMDS). The gut microbiota‐derived predicted microbial functional capacity was calculated using PICRUSt2 (Douglas et al., 2020).

### 2.7 Statistical analysis

All results are expressed as mean ± standard deviation. An analysis of variance with the Duncan test was performed to evaluate significant differences between each group. Analysis of Molecular Variance (AMOVA) was conducted to assess significant differences in the gut microbiota. Linear discriminant analysis effect sizes (LEfSe) were used to evaluate the OTUs and taxonomic categories of the gut microbiota with significant differences between groups (Segata et al., 2011). Welch's *t*‐test was performed to evaluate the predicted microbial functional capacity between various groups using STAMP software (Parks et al., 2014).

## 3 RESULTS

### 3.1 Polycan supplementation inhibited HFD‐induced weight gain

**FIGURE 1:**
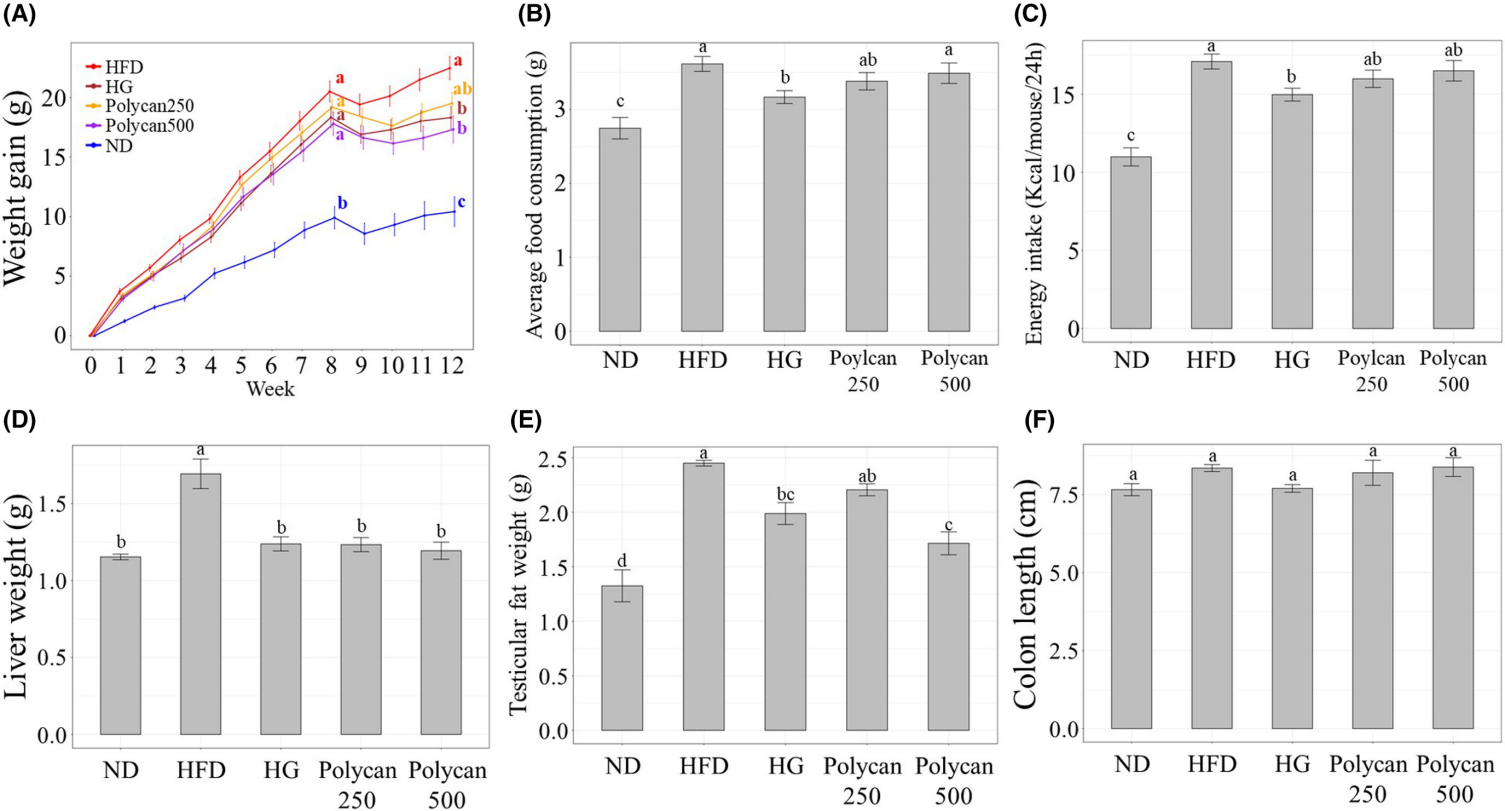
Effects of Polycan on mice on HFD. (A) Weight gain at 12 W, (B) average food consumption, (C) energy intake, (D) liver weight, (E) testicular fat weight, and (F) colon length (a > b > c > d; a significant difference at *p* < .05). Data are expressed as mean ± standard deviation (*n* = 8–9). HFD, high‐fat diet; HG, high‐fat diet with galacto‐oligosaccharide; ND, normal diet; Polycan250, high‐fat diet with Polycan (250 mg/kg); Polycan500, high‐fat diet with Polycan (500 mg/kg).

As shown in Figure 1A, during 8 weeks of consuming only HFD and ND, a significant difference in body weight gain was observed in mice (*p* < .05). However, GOS and Polycan supplementation for 4 weeks after 8 weeks had a lower final weight gain than the HFD group. In particular, the GOS and Polycan500 groups showed significantly lower final weight gain than the HFD group (*p* < .05). However, no significant difference was observed between the Polycan500 and Polycan250 groups. Interestingly, no significant difference was observed in food consumption and energy intake between the HFD and Polycan groups (Figure 1B,C). Furthermore, the liver and testicular fat weights of the mice on HFD were significantly higher than those on ND (*p* < .05). Conversely, supplementation with GOS and Polycan reversed this effect (Figure 1D,E). Compared with the HFD group (*p* < .05), the Polycan500 group showed significantly lower liver and testicular fat. However, the Polycan250 group only showed significantly lower liver weight (*p* < .05). Interestingly, Polycan supplementation decreased body and testicular weight dose‐dependently, except for liver weight. No significant differences were observed in colon length between groups (Figure 1F).

### 3.2 Polycan improved HFD‐induced colon damage

**FIGURE 2:**
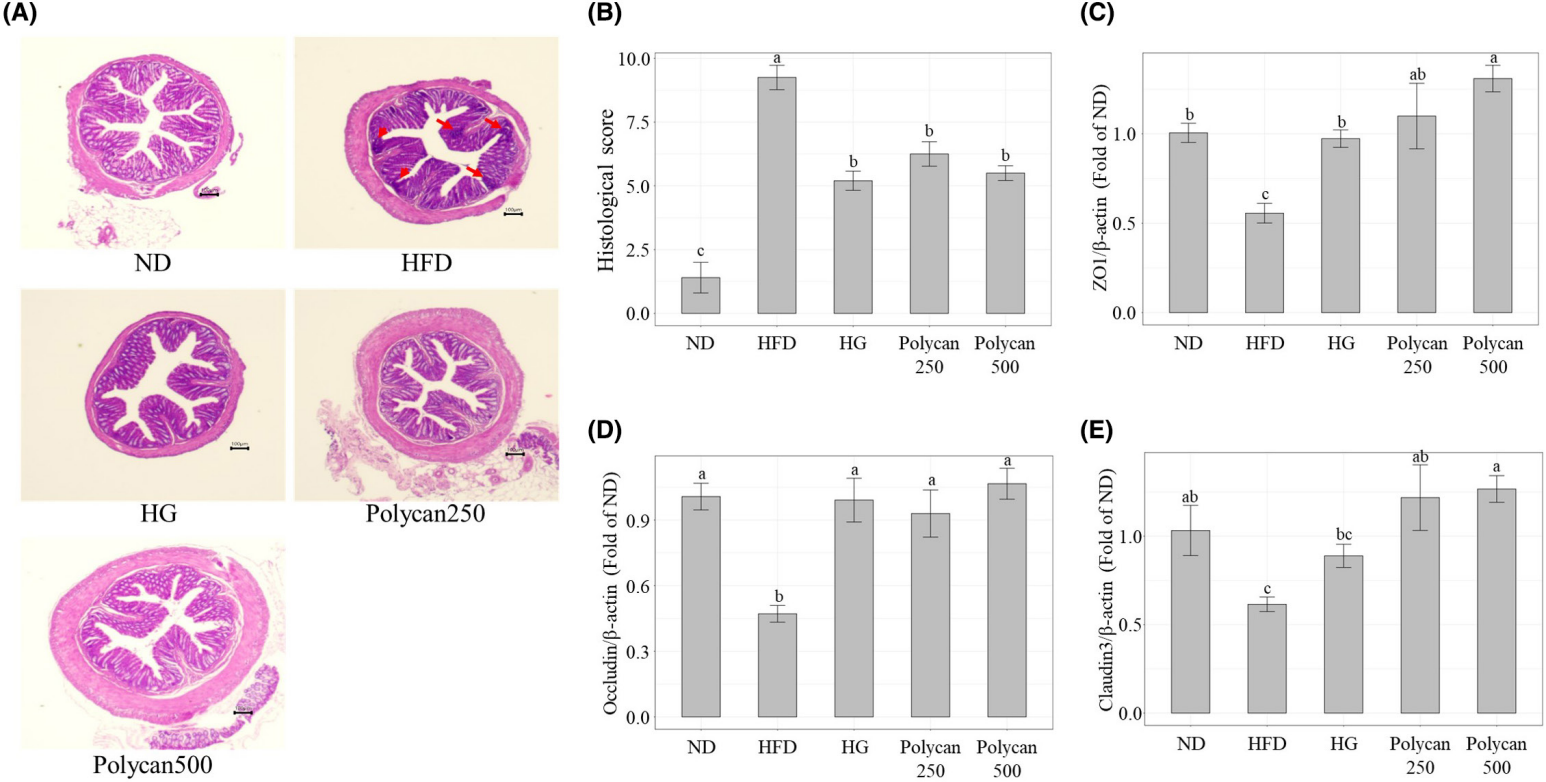
Effect of Polycan administration on the colons of high‐fat diet (HFD)‐induced obesity mice. (A) Hematoxylin and eosin staining of colon tissues (scale bar, 100 μm), (B) histological score, and (C–E) the mRNA expression of tight junction genes under the various dietary interventions (a > b > c > d; a significant difference at *p* < .05). Data are presented as mean ± standard deviation (*n* = 6). HFD, high‐fat diet; HG, high‐fat diet with galacto‐oligosaccharide; ND, normal diet; Polycan250, high‐fat diet with Polycan (250 mg/kg); Polycan500, high‐fat diet with Polycan (500 mg/kg).

We performed H&E staining to investigate the histological damage to colon tissue. As shown in Figure 2A, colon tissues from the HFD group showed inflammatory cell infiltration and decreased goblet cell numbers, which were alleviated by GOS and Polycan treatment. Likewise, the histological score of colons also showed that GOS and Polycan treatment mitigate colonic damage‐induced HFD (Figure 2B). In addition, we investigated the mRNA expression of TJ genes, such as *ZO1*, *OCLN*, and *CLDN3*, in mouse colon tissues. As shown in Figure 2C–E, TJ mRNA expression in the HFD group was significantly lower than that in the ND group (*p* < .05). The administration of GOS and Polycan significantly increased TJ mRNA expression (*p* < .05), but no significant difference was observed between the Polycan250 and Polycan500 groups.

### 3.3 Polycan supplementation modulated metabolite production by microbiota

To evaluate the effect of Polycan supplementation on gut microbial metabolites, fecal SCFA and phenolic and skatole concentrations were measured (Table 1). Compared with ND, HFD significantly reduced SCFA production (*p* < .05) and significantly increased the pH and production of harmful substances, such as phenol, *p*‐cresol, and skatole (*p* < .05). However, these trends were reversed by GOS and Polycan interventions. Acetate and butyrate, and thus the total SCFA production, were particularly dose‐dependently increased. However, dose‐dependent trends in the pH and production of phenol and skatole were not observed.

**TABLE 1:** Effects of Polycan on fecal microbial metabolites and pH.

Item	ND	HFD	HG	Polycan250	Polycan500
SCFA					
Acetate (μmol/g)	14.57 ± 1.35^a^	6.12 ± 0.1^d^	10.78 ± 0.49^b^	8.45 ± 0.67^c^	11.2 ± 0.41^b^
Propionate (μmol/g)	6.42 ± 0.42^a^	4.51 ± 0.04^c^	5.63 ± 0.16^b^	5.1 ± 0.1^bc^	5.77 ± 0.24^b^
Butyrate (μmol/g)	5.62 ± 0.19^a^	4.42 ± 0.07^d^	4.97 ± 0.23^b,c^	4.72 ± 0.08^c,d^	5.26 ± 0.19^a,b^
Total SCFA (μmol/g)	26.61 ± 1.87^a^	15.05 ± 0.15^d^	21.37 ± 0.58^b^	18.28 ± 0.74^c^	22.23 ± 0.69^b^
pH	7.88 ± 0.02^b^	8.06 ± 0.03^a^	7.76 ± 0.1^b^	7.94 ± 0.04a^b^	7.76 ± 0.05^b^
Phenolic and skatole compounds					
Phenol (μmol/g)	13.45 ± 2.71^b^	28.46 ± 7.17^a^	9.3 ± 1.13^b^	11.42 ± 0.67^b^	6.82 ± 1.76^b^
*p*‐Cresol (μmol/g)	13.71 ± 1.54^b^	43.43 ± 6.25^a^	16.21 ± 0.94^b^	12.88 ± 3.99^b^	10.1 ± 2.84^b^
Skatole (μmol/g)	0.88 ± 0.19^c^	2.39 ± 0.16^a^	1.33 ± 0.07^b^	1.15 ± 0.05^bc^	1.04 ± 0.12^bc^

*Note*: a > b > c > d; a significant difference at *p* < .05.

Abbreviations: HFD, high‐fat diet; HG, high‐fat diet with galacto‐oligosaccharide; ND, normal diet; Polycan250, high‐fat diet with Polycan (250 mg/kg); Polycan500, high‐fat diet with Polycan (500 mg/kg); SCFA, short‐chain fatty acids.

### 3.4 Polycan supplementation ameliorated HFD‐induced dysbiosis in the gut microbiota

**FIGURE 3:**
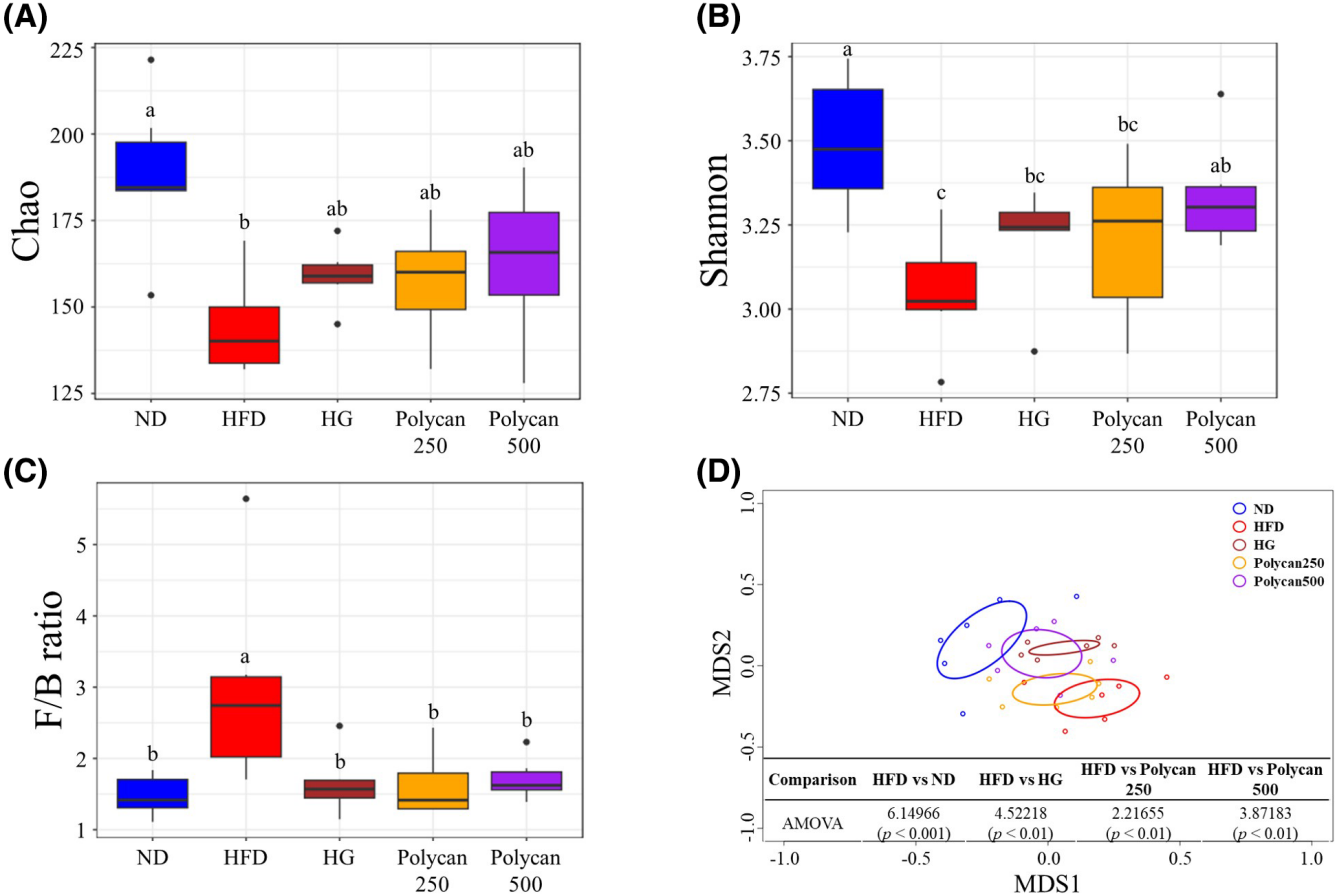
Comparison of gut microbiota using ecological indices, Firmicutes/Bacteriodetes (F/B) ratio, and nonmetric multidimensional scaling (NMDS). (A) Chao, (B) Shannon, (C) F/B ratio, and (D) NMDS with analysis of molecular variance (AMOVA). (a > b > c; a significant difference at *p* < .05). HFD, high‐fat diet; HG, high‐fat diet with galacto‐oligosaccharide; ND, normal diet; Polycan250, high‐fat diet with Polycan (250 mg/kg); Polycan500, high‐fat diet with Polycan (500 mg/kg).

The coverage used as an indicator for evaluating sequencing completeness was calculated as >99% for all samples (Figure S1). The Chao and Shannon indices, which indicate species richness and evenness, respectively, varied significantly between the ND and HFD groups (Figure 3A,B). In contrast, the GOS and Polycan groups showed an increasing tendency in their Chao and Shannon indices, which had been decreased by HFD. Of note, the ecological indices in the Polycan500 group were significantly higher than those in the HFD group. In addition, not only was the Firmicutes/Bacteriodetes (F/B) ratio for the HFD group significantly lower than those of the other groups, but NMDS and AMOVA showed significant differences between the HFD group and the other groups (Figure 3C,D). These results suggest that HFD induced dysbiosis in the mouse gut microbiota and that Polycan reversed the condition.

The taxonomic composition of fecal microbiota at the phylum, family, and genus levels was evaluated by determining the relative abundance, which is expressed as heatmaps in Figure S2. At the phylum level, Bacteroidetes members in the HFD group were significantly lower than those in the other groups. This is because of the significantly lower numbers of species under the genus *Phocaeicola* of the Family Bacteroidaceae and of members of the Family Muribaculaceae and Order Bacteroidales than those in the other groups. At the family level, GOS and Polycan administration reduced Atopobiaceae in HFD‐fed mice due to the reduction of *Olsenella* species at the genus level.

**FIGURE 4:**
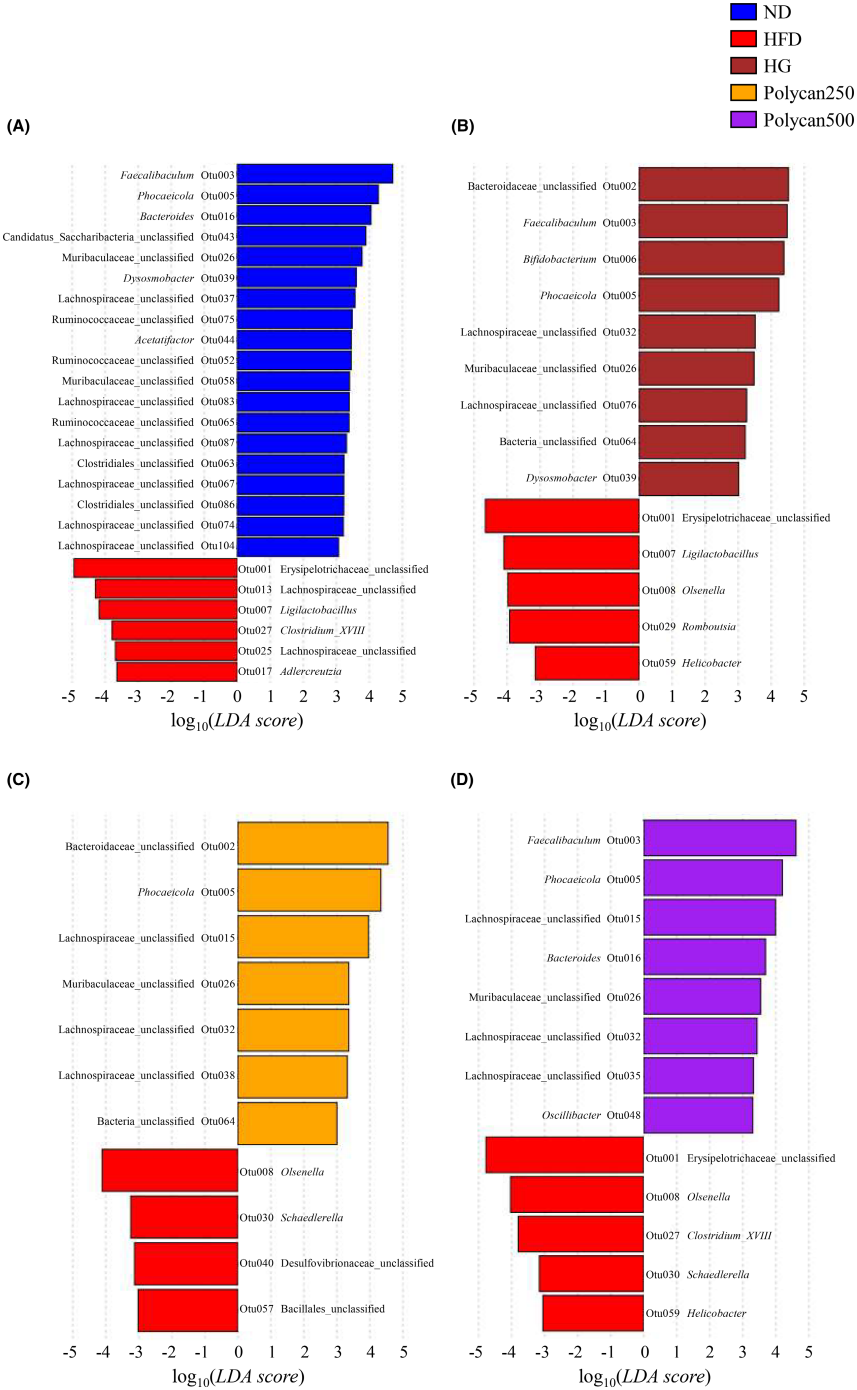
Effect of Polycan on the gut microbiota of mice fed a high‐fat diet (HFD). (A) HFD v/s CTL, (B) HFD v/s HG, (C) HFD v/s Polycan250 and (D) HFD v/s Polycan500. Significantly different relative abundance analyses were examined using linear discriminant analysis effect sizes at the OTU level (*p* < .05, LDA score > 3).

To provide a more detailed explanation of the changes in fecal microbiota at a lower taxonomic level induced by Polycan, OTUs that exhibited significant increases or decreases between the HFD group and other groups were identified using LEfSe. (Figure 4). HFD increased OTUs belonging to the unclassified Erysipelotrichaceae (Otu001), Lachnospiraceae (Otu013 and Otu025), and genera *Ligilactobacillus* (Otu007), *Adlercreutzia* (Otu017), and *Clostridium* XVIII (Otu027). Conversely, the following OTUs decreased: unclassified Muribaculaceae (Otu026 and Otu058); Candidatus Saccharibacteria (Otu043); Ruminococcaceae (Otu052, Otu062, and Otu075); Clostridiales (Otu063 and Otu086); Lachnospiraceae (Otu037, Otu067, Otu074, Otu083 Otu087, and Otu104); *Faecalibaculum* (Otu003); *Phocaeicola* (Otu005); *Bacteroides* (Otu016); *Alistipes* (Otu036); *Dysosmobacter* (out039); and *Acetatifactor* (Otu044). Among these OTUs, *Faecalibaculum* (Otu003), *Phocaeicola* (Otu005), *Bacteroides* (Otu016), Muribaculaceae (Otu026), *Alistipes* (Otu036), and *Dysosmobacter* (Otu039) increased in HFD‐fed mice under GOS or Polycan administration, whereas Erysipelotrichaceae (Otu001), *Ligilactobacillus* (Otu007), and *Clostridium* XVIII (Otu027) decreased. Additionally, GOS or Polycan administration increased the OTUS of the unclassified Bacteroidaceae (Otu002), Lachnospiraceae (Otu015, Otu032, Otu035, Otu038, and Otu076), *Bifidobacterium* (Otu006), and *Oscillibacter* (Otu048). Conversely, these two dietary interventions decreased the unclassified Desulfovibrionaceae (Otu040), Bacillales (Otu057), *Romboutsia* (Otu029), *Helicobacter* (Otu059), and *Schaedlerella* (Otu030). In particular, compared with that in the HFD group, Lachnospiraceae (Otu032) increased in all GOS, Polycan250, and Polycan500 groups, whereas *Olsenella* (Otu008) decreased.

### 3.5 Polycan administration altered the predicted microbial functional capacity

The prediction of microbial functional capacity based on the microbial community and the KEGG enzyme database was carried out using PICRUSt2. Figure 3 presents heatmaps of the enzymes in the KEGG pathway involved in the production of SCFA (pyruvate, propanoate, and butanoate metabolism) and phenolic and indolic compounds (tryptophan and tyrosine metabolism) that showed significant differences between the HFD and other groups. Thirty‐six enzymes were detected, most of which were enzymes involved in the SCFA metabolite pathway. Among these enzymes, 15 and 8 were decreased or increased, respectively, by HFD. Of these, succinate dehydrogenase, malate dehydrogenase, acetate‐CoA ligase, lactaldehyde dehydrogenase, and 2‐oxoacid oxidoreductase were higher in all other groups than in the HFD group. In addition, butyrate kinase, phosphate butyryltransferase, malate dehydrogenase, and pyruvate carboxylase increased due to GOS and Polycan administration, whereas aldehyde dehydrogenase, which is involved in SCFA and tryptophan metabolism, decreased.

## 4 DISCUSSION

Polycan is extracted from *A. pullulans* SM‐2001, a UV‐induced mutant of *A. pullulans*, and mostly contains β‐1,3/1,6‐glucans and amino acids, mono‐ or diunsaturated fatty acids (linoleic and linolenic acids), and fibrous polysaccharides (Kim et al., 2012). Thus, it exhibits properties that differ somewhat from those of other β‐glucans (Seo et al., 2002). Dysbiosis is defined as an imbalance in the bacterial composition. It is associated with many metabolic diseases, either by improving or damaging the intestinal barrier (DeGruttola et al., 2016; Zhang, Cheng, et al., 2021). Polycan has previously demonstrated therapeutic effects against several metabolic diseases (Kim et al., 2012, 2020; Lim et al., 2015). This may be attributed to the β‐glucan component and various organic substances comprising it that improve dysbiosis, thereby ultimately alleviating metabolic diseases (Ahmadi et al., 2017; Lee et al., 2019; Maguire & Maguire, 2019; Mo et al., 2022; Muthuramalingam et al., 2019; Tsutsumi et al., 2021). Therefore, we hypothesized that the beneficial effects of Polycan may be partially responsible for the reversal of dysbiosis and improvement of the intestinal barrier.

We investigated the efficacy of Polycan against dysbiosis in an HFD mouse model. In our study, HFD significantly affected the gut microbiota, which was reversed by Polycan administration toward normal levels in a dose‐dependent manner. Additionally, HFD increased the F/B ratio, but Polycan decreased it. The F/B ratio plays an important role in maintaining normal intestinal homeostasis, and its increase under obesity conditions is generally considered dysbiosis (Stojanov et al., 2020). In our study, the HFD group also showed greater body weight gain than the ND group, which was also dose‐dependently reduced by Polycan administration regardless of food intake. Overall, Polycan reversed dysbiosis and body weight gain in mice through beneficial changes in the gut microbiome.

A healthy‐functioning intestine plays a key role in the metabolism and distribution of nutrients, electrolytes, and fluids. Additionally, it is essential for preventing the entry of harmful substances from the intestinal lumen into the body (Konig et al., 2016; Vancamelbeke & Vermeire, 2017). Goblet cells are cells in the intestinal lining that secrete mucins, which prevent the gut microbiota and the chemicals they produce from entering the intestinal barrier (Kim & Khan, 2013). Epithelial cells are tightly bound together by TJ proteins that inhibit pathogen invasion (Bernardi et al., 2020). HFD destroys the intestinal epithelial barrier and TJ proteins, resulting in an inflammatory response that induces histological changes, such as the reduction of epithelial and goblet cells and inflammatory cell infiltration (Rohr et al., 2020). These histological changes and decreased mRNA expression of TJ genes (*ZO1*, *OCLDN*, and *CLDN3*) were observed in our study and were subsequently reversed by Polycan administration. In a previous study, our research group reported that Polycan administration alleviated the disruption of intestinal barrier integrity by increasing TJ‐related protein expression levels in the DSS colitis‐induced mouse model (Do et al., 2023). Thus, Polycan is a promising treatment option for intestinal barrier damage. Furthermore, intestinal barrier damage induced by HFD is associated with dysbiosis. HFD generally worsens gut health by reducing beneficial bacteria while increasing barrier‐destroying microbes (Jiang & Miao, 2023). In a previous study, the transfer of gut microbiota from mice induced with a HFD into a sterile mouse colon led to the development of a metabolic syndrome phenotype marked by epithelial barrier dysfunction, irrespective of the recipient diet (Bäckhed et al., 2004). HFD also damages the intestinal wall by increasing reactive oxygen species generated by the gut microbiota (Zeng et al., 2023).

SCFAs are fatty acids composed of <6 carbons, mainly acetate, propionate, and butyrate. They are produced by anaerobic bacteria in the large intestine. They confer beneficial effects on intestinal homeostasis and energy metabolism while exerting an anti‐inflammatory function in the intestinal mucosa. This is achieved through the activation of G‐protein‐coupled receptors in both intestinal epithelial cells and immune cells (van der Hee & Wells, 2021). Butyrate enhances intestinal barrier function by promoting the expression of TJ proteins. (Cani & Jordan, 2018). In addition, increased SCFA production increases the intestinal pH, which inhibits the growth of harmful bacteria (Den Besten et al., 2013). However, HFD reduces SCFA concentrations, as shown in several studies (Ilyes et al., 2022). The present study also confirms decreased fecal SCFA concentrations caused by HFD, which were subsequently reversed by Polycan administration. This may be due to the changes in the gut microbiota induced by Polycan. In HFD‐fed mice, Polycan administration increased the abundance of bacterial species under *Phocaeicola*, *Bacteroides*, Lachnospiraceae, and Muribaculaceae, which were more abundant in the ND group than in the HFD group. Previous studies have reported that many Lachnospiraceae and Muribaculaceae bacterial strains produce SCFAs from fibrous polysaccharides (Abdugheni et al., 2022; Smith et al., 2019). In particular, *Bacteroides* has polysaccharide utilization loci in its genome, which can directly metabolize β‐glucan and produce SCFAs (Fernandez‐Julia et al., 2021). Furthermore, in the present study, the predicted microbial functional capacity related to SCFA production increased. Lactaldehyde dehydrogenase and 2‐oxoacid oxidoreductase produce pyruvate, lactate, and acetyl‐CoA, which are precursors of SCFA (Zhang, Zuo, et al., 2021). Of these, pyruvate can produce propanoate via the succinate pathway. Pyruvate is sequentially converted by intestinal microorganisms into oxaloacetate, malate, fumarate, and succinate. Succinate is converted to propionyl‐CoA to produce propanoate (Connors et al., 2018). Pyruvate carboxylase (pyruvate to oxaloacetate), malate dehydrogenase (oxaloacetate to malate), malate dehydrogenase (pyruvate to malate), fumarate hydratase (malate to fumarate), succinate dehydrogenase (fumarate to succinate), and acetate‐CoA ligase (propionyl‐CoA to propanoate) were increased by Polycan administration. Interestingly, an increase in succinate pathway‐related and other enzymes was observed in *Bacteroides* (Louis & Flint, 2017; Reichardt et al., 2014). Meanwhile, phosphate butyryltransferase and butyrate kinase, which produce butanoate from butanoyl‐CoA, were also increased by Polycan administration in HFD‐fed mice. In addition, Polycan administration resulted in a dose‐dependent increase in SCFAs, which is due to an increase in *Faecalibaculum* and *Oscillibacter* strains, which are SCFA producers (Cao et al., 2022; Iino et al., 2007). These results suggest that changes in SCFA‐related bacteria caused by Polycan contribute to the recovery of SCFA production.

Polycan reduced the production of phenol, *p*‐cresol, and skatole, which weaken the intestinal barrier in HFD‐fed mice. Previous studies have shown that phenol can destabilize TJ‐containing microdomains and reduce intestinal barrier function (McCall et al., 2009), whereas *p*‐cresol inhibits the growth of colonic epithelial cells by inhibiting cellular ATP synthesis and increasing DNA damage (Andriamihaja et al., 2015). Furthermore, skatole increases TNF‐α and impairs the function of intestinal epithelial cells (Kurata et al., 2023). These substances are metabolites produced by the gut microbiota from aromatic amino acids derived from dietary proteins. The beneficial gut microbiota changes exhibited by Polycan included a decrease in *Olsenella*, *Clostridium* XVIII, and *Schaedlerella* strains that produce such detrimental substances (Saito et al., 2018; Zgarbová & Vrzal, 2022). Furthermore, Polycan administration decreased the predicted microbial functional capacity related to skatole production in HFD‐fed mice. Aldehyde dehydrogenase produces indole‐3‐acetate, the precursor of skatole, from indole‐3‐acetaldehyde. In particular, previous studies have reported that *Olsenella uli* and various *Clostridium* strains convert indole‐3‐acetate into skatole (Whitehead et al., 2008; Zgarbová & Vrzal, 2022). In addition, Olsenella is highly associated with dysbiosis and inflammation (Wang et al., 2022), and *Clostridium innocuum* not only causes diarrhea but is also abundant under intestinal inflammation conditions in Crohn's disease (Bhattacharjee et al., 2023; Ha et al., 2020). Thus, Polycan can improve HFD‐induced intestinal barrier damage by suppressing pathogenic bacterial activity.

This study has some limitations. Inflammation in the colon was confirmed only through H&E staining. More accurate results could have been obtained if enzyme‐linked immunosorbent assays or mRNA and protein expression analyses were performed. In addition, *Bacteroides* species, the activities of which are promoted by Polycan, also produce phenol, *p*‐cresol, and skatole (Saito et al., 2018; Zgarbová & Vrzal, 2022). Therefore, additional experiments should be conducted on the correlation between *Bacteroides* and the concentrations of phenolic and indolic compounds to clarify the contributions of the bacterial species to intestinal barrier damage. Nevertheless, these results provide information on the Polycan effect in mitigating dysbiosis by HFD. To our knowledge, this is a novel in vivo experimental study examining changes in microbial metabolites, including phenolic and indolic compounds, with improvements in intestinal barrier damage and linking the results to changes in the mice gut microbiota. Thus, it provides knowledge of improving gut barrier damage from the perspective of microbial metabolites and the shifting gut microbiota in the development of functional foods.

## 5 CONCLUSION

In conclusion, Polycan improved intestinal barrier damage and dysbiosis induced by HFD in mice. Polycan not only increased SCFA production but also increased the numbers of SCFA‐producing bacteria and SCFA‐related predicted microbial functional capacity. Furthermore, Polycan supplementation decreased the levels of toxic substances (phenol, *p*‐cresol, and skatole), as well as the bacterial populations producing these toxic substances and the related predicted microbial functional capacity. Our results further show that the health benefits of Polycan are partially due to changes induced in the gut microbiota, suggesting that Polycan can be used as a functional food supplement for promoting gut health.

## AUTHOR CONTRIBUTIONS

**Gwang‐Pyo Ko:** Conceptualization (lead); data curation (lead); formal analysis (lead); investigation (lead); methodology (lead); software (lead); validation (lead); visualization (lead); writing – original draft (lead); writing – review and editing (equal). **Tatsuya Unno:** Conceptualization (supporting); funding acquisition (supporting); project administration (supporting). **Young‐Suk Kim:** Funding acquisition (supporting); project administration (supporting); resources (lead). **Jungman Kim:** Conceptualization (lead); methodology (lead); project administration (lead); supervision (lead); validation (lead).

## FUNDING INFORMATION

This work was financially supported by the Basic Science Research Program through the National Research Foundation of Korea (NRF), funded by the Ministry of Education (2021R1I1A1A01057460, 2016R1A6A1A03012862).

## CONFLICT OF INTEREST STATEMENT

The authors have no conflict of interest to declare.

## ETHICS STATEMENT

The animal experiment was approved and conducted by the Institutional Animal Care and Use Committee at Jeju National University (Approval number 2022–0054).

## Supporting information


Table S1:

## Data Availability

The data that support the findings of this study are available from the corresponding author upon reasonable request.
