# Supplementary material for: Dietary Polycan, a β‐glucan originating from Aureobasidium pullulans SM‐2001, attenuates high‐fat‐diet‐induced intestinal barrier damage in obese mice by modulating gut microbiota dysbiosis
Source: Food Sci Nutr. 2024 May 26;12(8):5824–35. doi: 10.1002/fsn3.4235 (PMC11317661; doi:10.1002/fsn3.4235)
Supplement: Supplementary file 1 — Table S1: [file FSN3-12-5824-s001.pdf]

**Table S1. The compositions and formulas of normal diet and high-fat diet.**

| Class description | Ingredient (g/kg Diet) | Normal Diet g | Normal Diet Kcal | High-fat Diet g | High-fat Diet Kcal |
|---|---|---|---|---|---|
| Protein | Casein | 200 | 800 | 200 | 800 |
| Protein | L-Cystine | 3 | 12 | 3 | 12 |
| Carbohydrate | Corn Starch | 397.49 | 1589.9 | 72.8 | 291.2 |
| Carbohydrate | Dextrose | 132 | 528 | 100 | 400 |
| Carbohydrate | Sucrose | 100 | 400 | 172.8 | 691.2 |
| Fiber | Cellulose | 50 | 0 | 50 | 0 |
| Fat | Soybean Oil | 70 | 630 | 25 | 225 |
| Fat | Lard | 0 | 0 | 177.5 | 1597.5 |
| Mineral | Mineral mix | 35 | 0 | 45 | 0 |
| vitamin | Vitamin mix | 10 | 40 | 10 | 40 |
| vitamin | Choline Bitartrate | 2.5 | 0 | 2 | 0 |
| Anti-oxidant | t-BHQ | 0.01 | 0 | 0 | 0 |
| Total | | 1000 | 4000 | 858.1 | 4057 |
| **Component** | | **g (%)** | **Calorie (%)** | **g (%)** | **Calorie (%)** |
| Protein | | 20 | 20 | 24 | 20 |
| Carbohydrate | | 63 | 63 | 40 | 34 |
| Fat | | 7 | 16 | 24 | 45 |

**Table S2. Evaluation criteria of histological score of the colon**

| Feature Graded | Description | Grade |
|---|---|---|
| Normal tissue | None | 0 |
| Degree of epithelial surface damage | Local and slight | 1 |
| Degree of Crypt damage | Local and moderate | 2 |
| Degree of inflammatory factor infiltration | Local and severe | 3 |
| | Extensive and moderate | 4 |
| | Extensive and severe | 5 |

**Table S3. Primer information for real-time PCR.**

| Gene | | Primer (5' → 3') | Accession no. |
|---|---|---|---|
| ZO-1 | F | ACCCGAAACTGATGCTGTGGATAG | NM_009386.2 |
| | R | AAATGGCCGGGCAGAACTTGTGTA | |
| Occludin | F | ATGTCCGGCCGATGCTCTC | NM_008756.2 |
| | R | TTTGGCTGCTCTTGGGTCTGTAT | |
| Claudin 3 | F | CAGGGGCAGTCTCTGTGCGAG | NM_009902.4 |
| | R | GCCGCTGGACCTGGGAATCAAC | |
| β-Actin | F | GGTGGGAATGGGTCAGAAGG | NM_007393.5 |
| | R | CAGCACAGGGTGCTCCTC | |

**Table S4. GC conditions**

| Steps | Conditions | | |
|---|---|---|---|
| SCFA | | | |
| Column | DB-FFAP (30 m × 0.25 mm × 0.25 μm, Agilent) | | |
| Injection mode | Split mode (10:1 ratio) | | |
| Injection volume | 1 μl | | |
| Inlet temperature | 230°C | | |
| FID temperature | 280°C | | |
| Column oven temperature | Rate | Temperature | Hold time |
| | - | 80°C | 3 min |
| | 15°C/min | 200°C | 3 min |
| | 5°C/min | 230°C | 10 min |
| Phenol, *p*-cresol and skatole | | | |
| Column | DB-1 (30 m × 0.25 mm × 0.25 μm, Agilent) | | |
| Injection mode | Splitless mode | | |
| Injection volume | 2 μl | | |
| Inlet temperature | 280°C | | |
| FID temperature | 310°C | | |
| Column oven temperature | Rate | Temperature | Hold time |
| | - | 70°C | 5 min |
| | 5°C/min | 110°C | 2 min |
| | 6°C/min | 150°C | 2 min |
| | 20°C/min | 300°C | 2 min |

**Table S5. PCR conditions**

| Steps | Thermal parameters | Cycle number |
|---|---|---|
| First PCR (v3-v4) | 95°C, 3 min | - |
| | 95°C, 30sec; 55°C, 30sec; 72°C, 30sec | 25 |
| | 72°C, 5 min | - |
| | | |
| Second PCR (Index) | 95°C, 3 min | - |
| | 95°C, 30sec; 55°C, 30sec; 72°C, 30sec | 8 |
| | 72°C, 5 min | - |

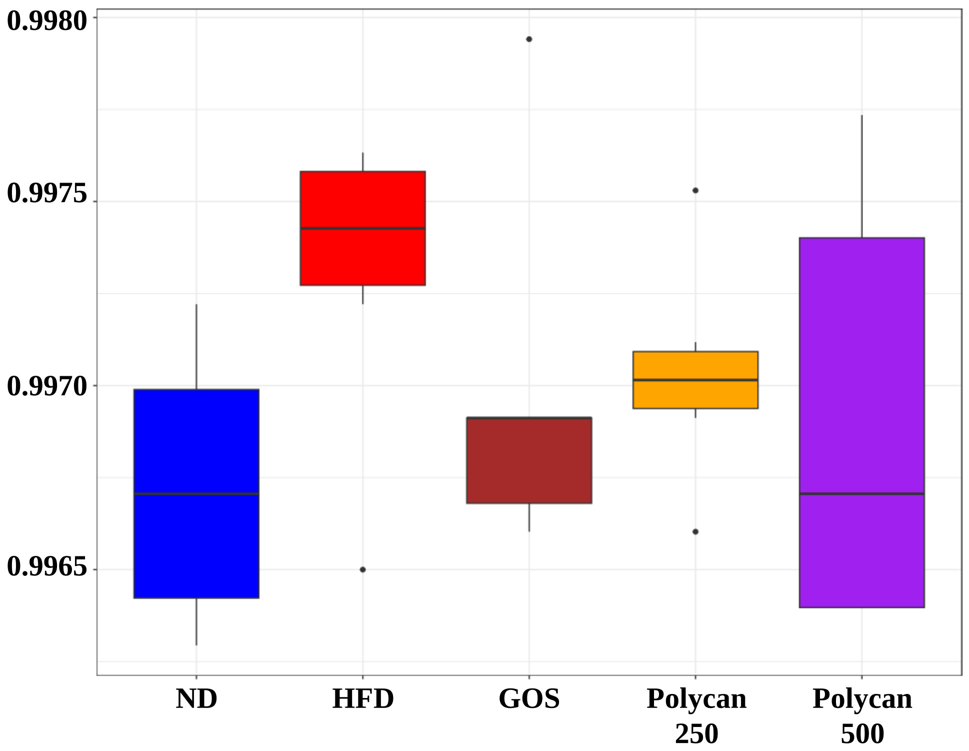


Fig. S1. Comparisons of the community Coverage.

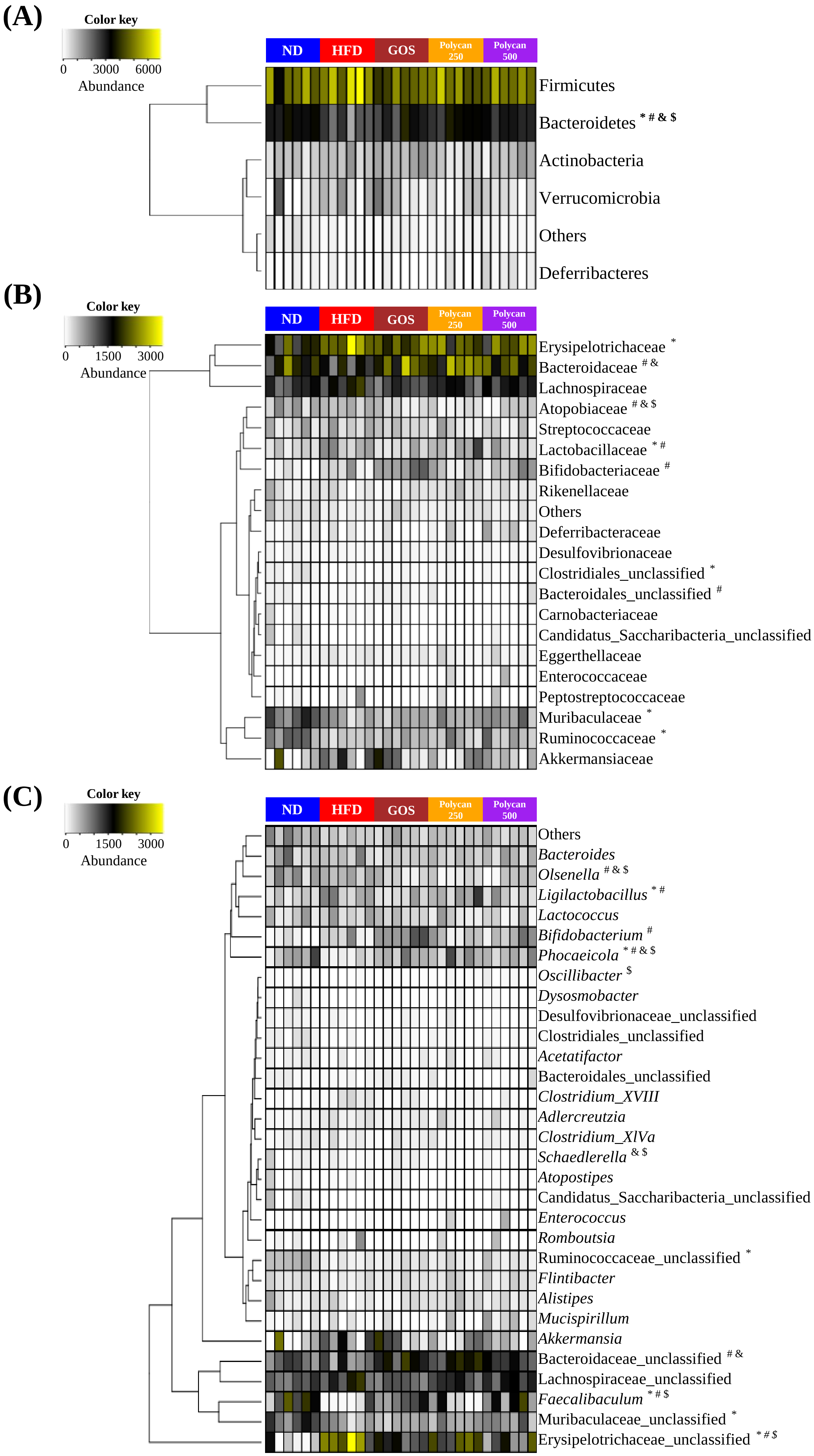


Fig. S2. Comparison of taxonomic composition. (A) Phylum, (B) Family, and (C) Genus. $^{*}$, $^{\#}$, $^{\&}$ and $^{\$}$ indicate significant difference between HFD and ND, HFD and HG, HFD and Polycan 250 and HFD and Polycan 500, respectively ($p < 0.05$). The relative abundance was examined using LEfSe analysis.

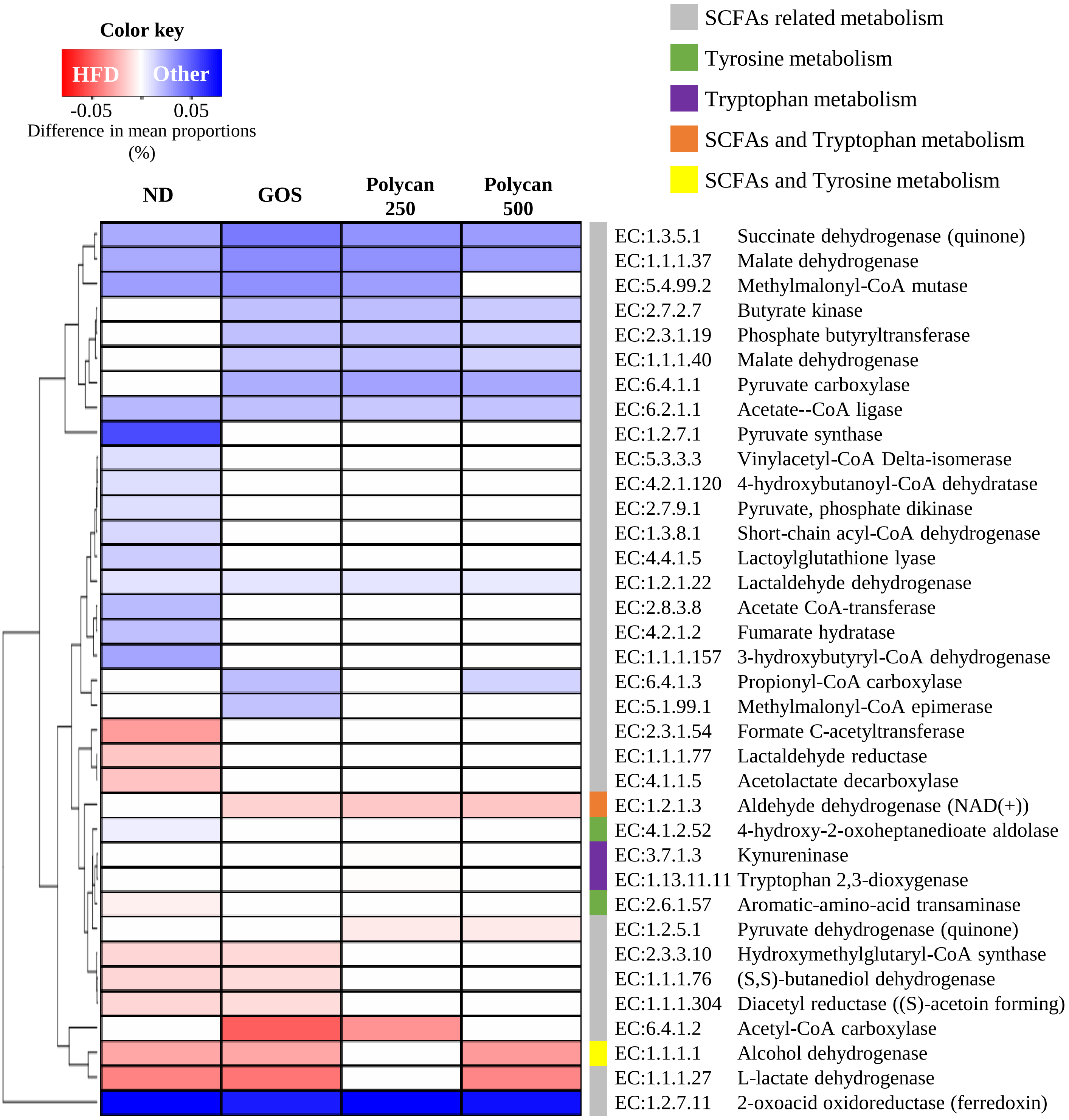


Fig. S3. Significant different relative abundance of PICRUSt2-predicted metabolic pathways between the comparing. ($P < 0.05$).
